# Optimizing risk stratification for intermediate-risk prostate cancer – the prognostic value of baseline health-related quality of life

**DOI:** 10.1007/s00345-024-05298-2

**Published:** 2024-10-20

**Authors:** Thilo Westhofen, Alexander Buchner, Simon Lennartz, Severin Rodler, Lennert Eismann, Can Aydogdu, Darjusch Askari-Motlagh, Elena Berg, Enya Feyerabend, Philipp Kazmierczak, Friedrich Jokisch, Armin Becker, Christian G. Stief, Alexander Kretschmer

**Affiliations:** 1 Department of Urology, Ludwig-Maximilians-University of Munich, Marchioninistrasse 15, 81377 Munich, Germany; 2 Institute for Diagnostic and Interventional Radiology, Faculty of Medicine, University Hospital Cologne, Cologne, Germany; 3 Institute for Diagnostic and Interventional Radiology, Ludwig-Maximilians-University of Munich, Munich, Germany

**Keywords:** Radical prostatectomy, Risk stratification, Intermediate-risk prostate cancer, Metastasis-free survival, EORTC QLQ-C30, Health-related quality of life

## Abstract

**Objective:**

To investigate the prognostic value of baseline health-related quality of life (HRQOL) for patients with intermediate-risk localized prostate cancer (IR-PCa) undergoing radical prostatectomy (RP).

**Methods:**

4780 patients with IR-PCa according to NCCN risk stratification were identified from a prospectively maintained database. All patients were treated with RP and had prospectively assessed baseline HRQOL. Main outcomes were oncologic endpoints metastasis-free survival (MFS); biochemical recurrence free survival (BRFS) and overall survival (OS). Multivariable Cox regression models assessed prognostic significance of baseline global health status (GHS) on survival outcomes. Harrell’s discrimination C-index was applied to calculate the predictive accuracy of the model. Decision curve analysis (DCA) tested the clinical net benefit associated with adding the GHS domain to our multivariable model (*p* < 0.05).

**Results:**

Median follow-up was 51 months. Multivariable analysis confirmed baseline GHS as an independent predictor for increased MFS (HR 0.976, 95%CI 0.96–0.99; *p* < 0.001), increased BRFS (HR 0.993, 95%CI 0.99–1.00; *p* = 0.027) and increased OS (HR 0.969, 95%CI 0.95–0.99; *p* = 0.002), indicating a relative risk reduction of 2.4% for MFS, 0.7% for BRFS and 3.1% for OS per 1-point increase of baseline GHS. Baseline HRQOL improved discrimination in predicting MFS, BRFS and OS. DCA revealed a net benefit over all threshold probabilities.

**Conclusions:**

We found baseline HRQOL to substantially improve risk stratification for the heterogeneous cohort of IR-PCa. Baseline HRQOL accurately predicts increased MFS, BRFS and OS. Our findings therefore support the role of preoperative HRQOL as an adjunct to established prognosticators for IR-PCa, potentially facilitating guidance of therapy.

**Supplementary Information:**

The online version contains supplementary material available at 10.1007/s00345-024-05298-2.

## Introduction

Intermediate-risk (IR) prostate cancer (PCa) represents a heterogenous subgroup of patients with variable tumor characteristics as well as oncological outcomes. Historically, the d’Amico risk classification has been used to define patients with IR-PCa [1]. Despite being only validated for biochemical recurrence, this classification is still in frequent use to date. To further account for the heterogeneity of IR-PCa patients, the NCCN [2] and AUA [3] classification further substratify IR-PCa into favourable or unfavourable disease, yet this substratification relies on similar stratification parameters as d’Amico. Consequently, substratification of IR-PCa patients is still subpar and next-generation imaging as well as genomic information, both frequently used in current day-to-day decision making, have not yet found their way into current classification systems [4].

In addition, several previous studies have demonstrated that health-related quality of life (HRQOL) can predict survival outcomes for several different cancer entities and thus pre-therapeutic HRQOL has become an increasingly recognised prognostic indicator of survival outcomes in various advanced and metastatic solid cancers [5–7] including high-risk localized prostate cancer [8]. The integration of preoperative patient-reported outcome measures (PROMs), namely HRQOL, into risk assessment enables a holistic view of the patient as well as the consideration of symptoms that might not be adequately captured by established clinicopathological parameters. However, there is no assessment of the prognostic accuracy of preoperative HRQOL assessment for IR-PCa patients to date.

## Materials and methods

### Patient population, study design and data assessment

Following approval by a local ethics committee (approval number of ethics committee #20-1022), 6487 patients from a prospective institutional database who underwent RP for PCa between January 2009 and December 2020 were identified. Surgical techniques in our department have been described previously [9]. 4780 patients met the inclusion criteria for the current study which encompassed: intermediate-risk localized PCa [as defined by National Comprehensive Cancer Network (NCCN) criteria [2]). Patients with incomplete data or lost to follow-up (*n* = 127) were excluded from further analysis (Suppl. Figure 1). Prospective assessment of HRQOL prior to surgery (baseline HRQOL) was performed using a validated translation of the standardised EORTC QLQ-C30 questionnaire [10]. Patients were stratified by baseline general HRQOL measured by the global health status domain (GHS) of the QLQ-C30 questionnaire, following EORTC instructions [11]. As per institutional standard of care, questionnaires were handed out to patients 1 to 3 days prior to RP.

### Outcomes

Primary endpoint was metastasis-free survival (MFS) based on conventional or PET-based imaging, which was calculated from date of the radical prostatectomy (RP). The secondary endpoints were biochemical recurrence free survival (BRFS) and overall survival (OS). Patients were censored at last follow-up including imaging or death.

### Follow up

Follow-up of eligible patients was performed at 3-month intervals within the first postoperative year, followed by annually intervals thereafter. Validated questionnaires were sent via mail to eligible patients. In addition, oncological outcome information was retrieved directly from patients, referring urologists, and primary physicians.

### Statistical analysis

For descriptive statistics, median and means were used to present continuous variables and percentages or absolute numbers to present non-continuous variables. Multivariable Cox regression models were used to examine the independent prognostic value of the GHS-domain of the EORTC QLQ-C30 questionnaire, stratified by preoperative clinicopathological variables clinical tumor stage (cT-stage), Gleason grade, PSA, age American Society of Anaesthesiologists physical status classification system (ASA-Score) and Charlson Comorbidity Index (CCI), which have previously shown to be relevant confounders [12, 13]. Multicollinearity was examined using Variance Inflation Factor (VIF) to ensure variable independence. If a VIF-values exceeds 10, this would be a strong indication of multicollinearity and the corresponding variable must be excluded [14]. Assumptions for Cox regression models were tested applying Schoenfeld residuals.

To assess the prognostic value of baseline HRQOL data, Harrell’s concordance index (C-index) was used to estimate the discrimination of our Cox regression models with and without GHS. The C-index estimates the proportion in which predicted survival and observed survival are concordant for all pairwise patient combinations of the dataset. A C-index of 0.5 represents random predictions, a C-index of 1.0 indicates a perfectly discriminating model. Decision curve analysis (DCA) was used to determine the clinical net benefit associated with adding GHS to our multivariable model compared to the multivariable model without GHS. A p-value of < 0.05 was considered statistically significant. Statistical analysis was performed using MedCalc Statistical Software version 20.011 (MedCalc Software, Belgium) and R software environment for statistical computing and graphics (version 4.1.3; R Foundation for Statistical Computing, Austria).

## Results

### Perioperative patient characteristics

A total number of 4780 patients fulfilled the inclusion criteria of the current study and were included for further analysis. Patient characteristics are provided in Suppl. Table 1. *N* = 2024 underwent preoperative staging examinations, of which 421 underwent PSMA PET prior RP. According to NCCN-classification, 1387 (29.0%) were favourable intermediate-risk and 3393 (71.0%) unfavourable intermediate-risk. Overall, 4470 patients have been followed without event with a median follow-up of 51 months (IQR 37–96).

### Survival of patients stratified by global health status

At the time of analysis, 142 patients had experienced MFS with a median time-to-event of 23.5 months. 5-yr-MFS estimates for the cohort were 93%. 5-yr-BRFS estimates for the cohort were 82%, 5-yr-OS estimates were 97%. There were no perioperative deaths. Suppl. Figures 2, 3 and 4 descriptively displays the Kaplan-Meier plots for MFS, BRFS and OS, stratified by baseline GHS quartiles.

### Impact of preoperative GHS on survival outcomes

Table 1 displays the results of the multivariable Cox regression analysis for MFS as well as BRFS and OS. Hereby, baseline GHS was confirmed as an independent predictor for increased MFS (HR 0.976 per 1-point increase of baseline GHS, 95%CI 0.96–0.99; *p* < 0.001).

Equally, baseline GHS was confirmed as an independent predictor for increased BRFS (HR 0.993, 95%CI 0.99–1.00; *p* = 0.027) and OS (HR 0.969, 95%CI 0.95–0.99; *p* = 0.002). The VIF-values for all variables ranged between 1.044 and 2.863, demonstrating no multicollinearity (Suppl. Table 1).

From a clinical point of view, these hazard ratios indicate that each 1-point increase of baseline GHS converts into a relative risk-reduction of 2.4% for MFS, 0.7% for BRFS and 3.1% for OS (Table 1). Those results held true in subgroup-analysis stratified into favourable and unfavourable intermediate risk PCa (Suppl. Table 3).

**Table 1:** Multivariable cox regression analysis regarding the endpoint MFS (metastasis-free survival) and OS (overall survival). (GHS = global health status, BMI = body mass index, CCI = Charlson comorbidity index ASA-score = American society of anesthesiologists physical status classification system)

Multivariate cox regression analysis of BRFS, MFS & OS for GHS				
BRFS (biochemical recurrence free survival)				
Parameter	HR	95% CI		*p* value
		**Lower**	**Upper**	
Baseline GHS	0.993	0.99	1.00	***0.027***
Favourable intermediate risk [y/n]	0.913	0.64	1.31	0.622
cT-stage [T2a/2b vs. T2c]	1.109	0.86	1.44	0.433
ISUP grade - biopsy [1 vs. 2/3]	1.191	1.08	1.32	***0.001***
iPSA [ng/ml]	1.041	1.01	1.07	***0.010***
Age [yrs]	1.029	0.96	1.11	0.436
ASA-Score	0.828	0.66	1.03	0.096
CCI	1.114	0.94	1.32	0.219
**MFS (metastasis free survival)**				
Baseline GHS	0.976	0.96	0.99	***< 0.001***
Favourable intermediate risk [y/n]	0.680	0.16	2.89	0.601
cT-stage [T2a/2b vs. T2c]	1.310	0.71	2.42	0.388
ISUP grade - biopsy [1 vs. 2/3]	1.246	0.99	1.56	0.057
iPSA [ng/ml]	1.062	0.99	1.13	0.074
Age [yrs]	1.184	0.98	1.44	0.085
ASA-Score	0.807	0.50	1.31	0.385
CCI	0.673	0.40	1.12	0.127
**OS (overall survival)**				
Baseline GHS	0.969	0.95	0.99	***0.002***
Favourable intermediate risk [y/n]	1.232	0.26	5.79	0.792
cT-stage [T2a/2b vs. T2c]	1.262	0.46	3.43	0.649
ISUP grade - biopsy [1 vs. 2/3]	1.248	0.86	1.82	0.248
iPSA [ng/ml]	1.133	1.04	1.23	***0.004***
Age [yrs]	0.974	0.76	1.26	0.840
ASA-Score	1.059	0.83	1.35	0.642
CCI	1.427	0.86	2.36	0.165

### Added prognostic value of baseline HRQOL

To calculate the prognostic value of baseline HRQOL assessment, we applied the C-index to estimate the discrimination and impact of adding baseline HRQOL to clinicopathological and sociodemographic data into our multivariable model. The multivariable Cox regression model including GHS-score resulted in higher discrimination predicting MFS (C-index 0.64, 95%CI 0.55–0.74) compared to multivariable Cox regression models limited to clinicopathological variables (C-index 0.59, 95%CI 0.53–0.69). Equally, the addition of GHS to our multivariable Cox regression model resulted in higher discrimination predicting BRFS [C-index 0.69, 95%CI 0.63–0.87 vs. 0.66, 95%CI 0.57–0.81)] and OS [C-index 0.73 (95%CI 0.60–0.87) vs. 0.69 (95%CI 0.55–0.83)] (Table 2).

**Fig. 1:**
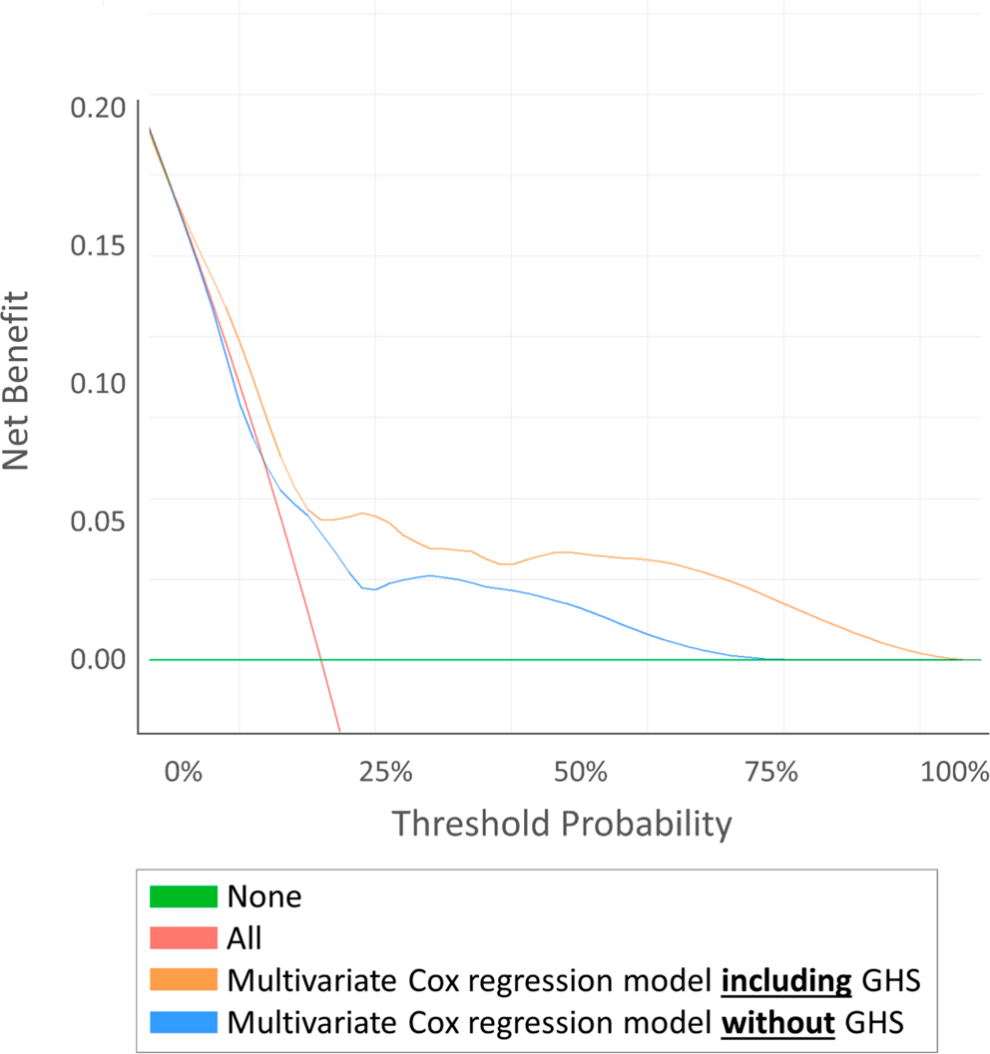
Decision curve analysis testing the clinical net benefit of adding GHS to our multivariable model in comparison with to the multivariable model without GHS in predicting the risk of distant metastasis at 51mo

**Fig. 2:**
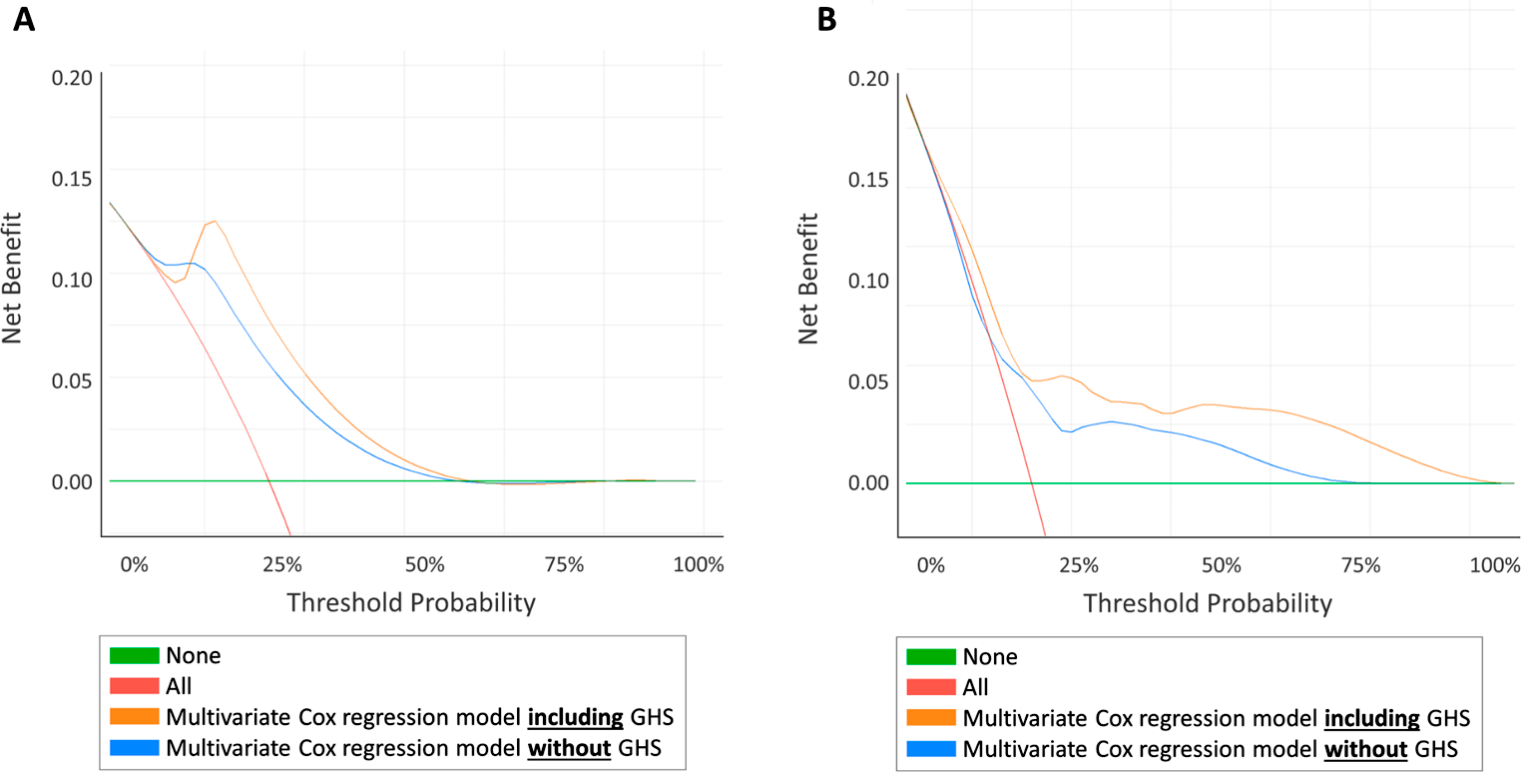
Decision curve analysis testing the clinical net benefit of adding GHS to our multivariable model in comparison with to the multivariable model without GHS in predicting (**A**) biochemical recurrence or (**B**) death at 51mo

DCA revealed that adding GHS to our multivariable model improved clinical risk prediction of MFS (Fig. 1), BRFS (Fig. 2A) and of OS (Fig. 2B) compared to the multivariable model without GHS throughout all threshold probabilities.

**Table 2:** Comparison of multivariable Cox regression model with and without GHS in predicting MFS and OS

Comparison of risk models in predicting BRFS, MFS & OS			
BRFS (biochemical recurrence free survival)			
Risk models	c-index	95% CI	
		Lower	Upper
multivariate Cox regression model **including** GHS	0.69	0.63	0.87
multivariate Cox regression model **without** GHS	0.66	0.57	0.81
**MFS** (metastasis-free survival)			
multivariate Cox regression model **including** GHS	0.64	0.55	0.74
multivariate Cox regression model **without** GHS	0.59	0.53	0.69
**OS** (overall survival)			
multivariate Cox regression model **including** GHS	0.73	0.6	0.87
multivariate Cox regression model **without** GHS	0.69	0.55	0.83

## Discussion

IR-PCa represents a very heterogeneous disease stage and it has been shown that patients with unfavourable disease characteristics based on NCCN criteria have a higher risk of impaired oncological outcomes following RP [15]. However, current risk stratification systems are insufficient [2–4]. Highly focused on histopathological and biochemical features, they do not reflect the vast heterogeneity of IR-PCa, underscoring the need for further refinement of currently available risk assessment tools.

The present study is the first analysis to show a significant prognostic value of preoperative baseline PROMs based on the validated EORTC QLQ-C30 questionnaire in this setting. In this large cohort of contemporary patients meeting intermediate-risk criteria [2], we found preoperative baseline general HRQOL assessed by the GHS-domain of the EORTC QLQ-C30 to independently predict BRFS, MFS and OS. Those results were confirmed in multivariable analysis which showed increased baseline GHS to be an independent predictor of prolonged BRFS, MFS and OS. Furthermore, those results held true in subgroup-analysis when favourable and unfavourable IR-PCa were assessed. Importantly, it has to be pointed out that GHS was assessed as a continuous variable. From a clinical implications point of view, the HR of 0.972 for MFS reflects that for each 1-point increase of pre-RP baseline GHS-scores, the likelihood of distant metastasis within the observed time period is reduced by 2.8%. Accordingly, the HR of 0.970 for OS reflects a 3.0% lower likelihood to experience death within the observed time period. In line with previous studies [8, 16], our results therefore demonstrate, that a better baseline HRQOL is a potential surrogate for better oncological outcomes.

As many IR-PCa patients harbour the risk for adverse pathology, the refinement of risk stratifications is of utter importance [17]. Neoadjuvant therapy with novel hormonal agents (NHT) could be a valuable therapy option for IR-PCa patients at higher risk for recurrence or metastasis. Previous studies however failed to show an oncological benefit of neoadjuvant NHT-therapy prior RP in unselected IR-PCa patients [18]. The implementation of baseline HRQOL in IR-PCa risk stratification might help to further identify patients who benefit from neoadjuvant treatment. The results of the current study are in line with previous studies reporting HRQOL parameters assessed prior treatment to add valuable prognostic strength in the prediction of survival for patients with advanced and metastatic stages of various cancer entities [5–7]. Furthermore, we have recently shown that baseline HRQOL can be a valuable and robust prognostic factor for patients with localized high-risk PCa prior RP and that baseline HRQOL could increase the prognostic accuracy of BRFS and MFS [8].

Regarding cancer entities other than PCa, a significant correlation between pre-treatment HRQOL and overall survival has been shown [5–7, 19, 20]. Hereby, more evidence has been generated for advanced disease stages with high symptom burden [7], and data for localized disease is still limited [21, 22]. In the largest analysis on the prognostic value of baseline HRQOL assessment for survival outcomes to date, Quinten et al. demonstrated that baseline HRQOL independently predicts OS in a multivariable analysis in a pooled dataset of 10,108 patients with 11 different advanced cancer entities [5]. Similar to these results, we confirmed the significant prognostic value of baseline HRQOL for IR-PCa in multivariable analysis stratified for previously defined confounders [12, 13]. Notably, we adjusted our analysis by ASA-score, an established measure for physical functioning, which was previously shown to be a predictor of survival outcomes following RP [13]. We furthermore sought to eliminate patients’ comorbidities as a potential confounder adjusting our analysis by validated CCI and consequently minimize potential selection bias. These results further underscore the hypothesis that increased baseline HRQOL assessed prior RP can predict improved BRFS, MFS and OS in IR-PCa patients.

Several attempts have been proposed to optimize risk stratification for localized PCa patients and to improve prognostic utility of establishes nomograms, measured by Harrell’s c-index [4, 23]. Hereby, one potential mitigation strategy focuses on the implementation of sophisticated next-generation imaging such as multiparametric MRI or PSMA-PET or molecular biomarkers into the pre-therapeutic risk assessment.

The inclusion of multiparametric MRI could be shown to improve prognostic accuracy of biochemical recurrence free survival, compared to available preoperative risk tools, in a cohort of low-, intermediate-, and high-risk PCa patients [24]. Klein et al., however, could show genomic classifiers to improve the predictive accuracy of metastasis free survival in high-risk PCa patients by 8% compared to commonly applied nomograms, ultimately reaching a c-index of 0.79 [25]. While clinically valuable if correctly used, these modalities have limitations in terms of availability as well as the financial burden for the patient or the respective healthcare system. Also, evidence on the predictive value of genomic classifiers or next-generation imaging to substratify IR-PCa is missing. Notably, the high variability in outcomes of the intermediate risk population represents a challenge, reflected by a low performance of established nomograms [26]. In the current IR-PCa cohort, we found baseline HRQOL to improve predictive accuracy of the prognosis for distant metastasis by 10% (c-index 0.64 vs. 0.59), for BRFS by 6% (c-index 0.69 vs. 0.66) and for overall survival by 8% (c-index 0.73 vs. 0.69). These findings were confirmed via DCA (Figs. 3 and 4), as the addition of baseline GHS to our multivariable model demonstrated a higher net benefit regardless of the baseline MFS, BRFS or OS threshold probability.

The assessment of baseline HRQOL implies various potential advantages. It has been shown that increased HRQOL is linked to improved treatment adherence and healthy behaviour [27]. This might have a conceivable positive impact on survival outcomes. Another possible explanation might be an earlier detection of symptom-deterioration by symptom domains of HRQOL questionnaires compared to conventional clinical measures. This has previously been shown for patients with colorectal cancer [28]. As PROMs ask different questions compared to traditional clinical measures and apply more sensitive scales than performance status scales, they might reflect functional aspects more reliably, which are closely related to survival. Previous studies have postulated a discrepancy between PROMS and clinical measures which might explain the added prognostic value [29, 30].

The current study supports the role of preoperative HRQOL assessment as an adjunct to established prognosticators. Especially since baseline HRQOL assessment is non-invasive, easy and unexpensive to implement and globally applicable with numerous validated translations of the EORTC QLQ-C30, it should be adopted in clinical practice at a low threshold to assist physicians in guidance of therapy.

Our study has several limitations, which are mainly inherent to the retrospective nature of the study. A potential patient selection bias typical for retrospective studies can therefore not be completely negated. The validation of our model in a prospective multicentric cohort could furthermore generate valuable information on the proportion of additional events detected by the addition of GHS into the model. In addition, assessment of HRQOL relied on the validated QLQ-C30 questionnaire, which is frequently used throughout oncologic trials and daily clinical routine assessments, but is not PCa-specific. Lastly, although our study lacks data on PCa-specific mortality, a recent meta-analysis could show MFS to meet the standards for valid surrogate parameters for PCa-specific survival and has consequently been included as the primary endpoint in the current study [31].

## Conclusion

Our findings highlight preoperative baseline general HRQOL assessed by EORTC Global Health Status to be a valuable and robust prognostic factor for patients with localized IR-PCa prior RP.

## Electronic supplementary material

Below is the link to the electronic supplementary material.

Supplementary Material 1

Supplementary Material 2

Supplementary Material 3

Supplementary Material 4

Supplementary Material 5

Supplementary Material 6

Supplementary Material 7

Supplementary Material 8

## Data Availability

Data is available for bonafide researchers on request from the corresponding author.
