# Supplementary material for: Optimizing risk stratification for intermediate-risk prostate cancer – the prognostic value of baseline health-related quality of life
Source: World J Urol. 2024 Oct 20;42(1):585. doi: 10.1007/s00345-024-05298-2 (PMC11491415; doi:10.1007/s00345-024-05298-2)
Supplement: Supplementary file 3 — Supplementary Material 3 [file 345_2024_5298_MOESM3_ESM.docx]

| Baseline characteristics | |
| --- | --- |
|  |  |
| No. of patients | 4780 |
| NCCN-classification[n (%)] |  |
| Favorable intermediate risk | 1387 (29.0) |
| Unfavorable intermediate risk | 3393 (71.0) |
| Baseline Global Health Status [median,IQR] | 75.0 [66.7, 83.3] |
| Age, yrs [median,IQR] | 65 [60, 70] |
| BMI kg/m2 [median,IQR] | 26.2 [24.3, 28.7] |
| PSA preop. ng/ml [median,IQR] | 6.9 [5.1, 9.8] |
| cT-stage[n (%)] |  |
| 2a/2b | 3560 (74.5) |
| 2c | 1220 (25.5) |
| ISUP grade - biopsy [n (%)] |  |
| 1 | 1912 (40.0) |
| 2 | 1563 (32.7) |
| 3 | 1305 (27.3) |
| Percent of positive biopsy cores [median,IQR] | 30.0 [16.7, 50.0] |
| ASA-score[n (%)] |  |
| 1 | 3550 (72.9) |
| 2 | 1023 (21.0) |
| 3 | 164 (3.4) |
| 4 | 43 (0.9) |
| Charlson comorbidity index (CCI) |  |
| 0 | 157 (3.3) |
| 1 | 923 (19.3) |
| 2 | 2162 (45.2) |
| 3 | 1214 (25.4) |
| 4 | 257 (5.4) |
| 5 | 48 (1.0) |
| 6 | 19 (0.4) |
| Prostate volume ml [median,IQR] | 50.2 [40.5, 64] |
| pT stage [n (%)]# |  |
| pT2a | 324 (6.8) |
| pT2b | 133 (2.8) |
| pT2c | 4323 (90.4) |
| ISUP grade - specism [n (%)] |  |
| 1 | 1338 (28.0) |
| 2 | 2144 (44.8) |
| 3 | 1298 (27.2) |
| Lymph node involvement [n (%)] | 67 (1.4) |
| Positive surgical margin [n (%)] | 636 (13.3) |
| Post-RP PSA-persistance [n (%)] [n (%)] | 292 (6.1) |
| Postoperative radiotherapy [n (%)] | 1109 (23.2) |
| Robot-assisted laparoscopic RP [n (%)] | 1068 (22.3) |
| Nerve sparing approach [n (%)] | 4254 (89.0) |

**Suppl. Table 1**. Patient characteristics of the patient cohort assessed in the current study (NCCN = National Comprehensive Cancer Network, BMI = body mass index, ASA-score = American Society of Anesthesiologists physical status classification system).
