# Supplementary material for: Optimizing risk stratification for intermediate-risk prostate cancer – the prognostic value of baseline health-related quality of life
Source: World J Urol. 2024 Oct 20;42(1):585. doi: 10.1007/s00345-024-05298-2 (PMC11491415; doi:10.1007/s00345-024-05298-2)
Supplement: Supplementary file 4 — Supplementary Material 4 [file 345_2024_5298_MOESM4_ESM.docx]

**A**

|  | **MFS (metastasis free survival)** | |  |
| --- | --- | --- | --- |
|  | **Parameter** | **VIF** |  |
|  | Baseline GHS | 1.208 |  |
|  | Favourable intermediate risk [y/n] | 1.095 |  |
|  | cT-stage | 1.047 |  |
|  | Gleason-grade biopsy | 1.117 |  |
|  | iPSA | 1.094 |  |
|  | Age | 2.524 |  |
|  | ASA-Score | 1.203 |  |
|  | CCI | 2.584 |  |
|  |  |  |  |

**B**

|  | **BRFS (Biochemical recurrence free survival)** | | |
| --- | --- | --- | --- |
|  | **Parameter** | **VIF** |  |
|  | Baseline GHS | 1.100 |  |
|  | Favourable intermediate risk [y/n] | 1.128 |  |
|  | cT-stage | 1.038 |  |
|  | Gleason-grade biopsy | 1.103 |  |
|  | iPSA | 1.077 |  |
|  | Age | 2.826 |  |
|  | ASA-Score | 1.142 |  |
|  | CCI | 2.863 |  |
|  |  |  |  |

**C**

|  | **OS (overall survival)** | |  |
| --- | --- | --- | --- |
|  | **Parameter** | **VIF** |  |
|  | Baseline GHS | 1.085 |  |
|  | Favourable intermediate risk [y/n] | 1.130 |  |
|  | cT-stage | 1.044 |  |
|  | Gleason-grade biopsy | 1.132 |  |
|  | iPSA | 1.089 |  |
|  | Age | 2.763 |  |
|  | ASA-Score | 1.119 |  |
|  | CCI | 2.795 |  |
|  |  |  |  |

**Suppl. Table 2**. Test for multicollinearity of independent variables (VIF= Variance Inflation Factor, GHS= global health status, PR = radical prostatectomy, PSA = prostate specific antigen, CCI = Charlson comorbidity index, ASA-score = American Society of Anesthesiologists physical status classification system)
