# Supplementary material for: Optimizing risk stratification for intermediate-risk prostate cancer – the prognostic value of baseline health-related quality of life
Source: World J Urol. 2024 Oct 20;42(1):585. doi: 10.1007/s00345-024-05298-2 (PMC11491415; doi:10.1007/s00345-024-05298-2)
Supplement: Supplementary file 5 — Supplementary Material 5 [file 345_2024_5298_MOESM5_ESM.docx]

|  | **favourable** | | | | |  | **unfavourable** | | | | |  |
| --- | --- | --- | --- | --- | --- | --- | --- | --- | --- | --- | --- | --- |
|  | **BRFS (biochemical recurrence free survival)** | | | | | | | | | | |  |
|  | **Parameter** | **HR** | **95% CI** | | **p value** |  | **Parameter** | **HR** | **95% CI** | | **p value** |  |
|  |  |  | **Lower** | **Upper** |  |  |  |  | **Lower** | **Upper** |  |  |
|  | Baseline GHS | 0.992 | 0.97 | 1.00 | ***0.043*** |  | Baseline GHS | 0.993 | 0.99 | 1.00 | ***0.036*** |  |
|  | cT-stage | 1.536 | 0.70 | 3.39 | 0.287 |  | cT-stage | 1.071 | 0.82 | 1.41 | 0.620 |  |
|  | Gleason-grade biopsy | 0.999 | 0.71 | 1.40 | 0.997 |  | Gleason-grade biopsy | 1.216 | 1.09 | 1.35 | ***<0.001*** |  |
|  | iPSA | 1.231 | 0.89 | 1.69 | 0.201 |  | iPSA | 1.039 | 1.01 | 1.07 | ***0.016*** |  |
|  | Age | 1.114 | 0.97 | 1.28 | 0.121 |  | Age | 1.026 | 0.95 | 1.11 | 0.517 |  |
|  | ASA-Score | 0.965 | 0.52 | 1.81 | 0.912 |  | ASA-Score | 0.814 | 0.64 | 1.04 | 0.093 |  |
|  | CCI | 1.261 | 0.77 | 2.07 | 0.361 |  | CCI | 1.105 | 0.92 | 1.33 | 0.288 |  |
|  | **MFS (metastasis free survival)** | | | | | | | | | | |  |
|  | **Parameter** | **HR** | **95% CI** | | **p value** |  | **Parameter** | **HR** | **95% CI** | | **p value** |  |
|  |  |  | **Lower** | **Upper** |  |  |  |  | **Lower** | **Upper** |  |  |
|  | Baseline GHS | 0.997 | 0.98 | 0.99 | ***0.029*** |  | Baseline GHS | 0.975 | 0.96 | 0.99 | ***<0.001*** |  |
|  | cT-stage | 1.063 | 0.70 | 1.33 | 0.533 |  | cT-stage | 1.410 | 0.75 | 2.63 | 0.281 |  |
|  | Gleason-grade biopsy | 1.496 | 0.83 | 1.75 | 0.153 |  | Gleason-grade biopsy | 1.270 | 1.01 | 1.60 | ***0.043*** |  |
|  | iPSA | 1.095 | 0.86 | 1.66 | 0.249 |  | iPSA | 1.069 | 1.00 | 1.14 | ***0.047*** |  |
|  | Age | 1.045 | 0.98 | 1.15 | 0.940 |  | Age | 1.216 | 0.99 | 1.49 | 0.059 |  |
|  | ASA-Score | 0.750 | 0.61 | 1.17 | 0.934 |  | ASA-Score | 0.792 | 0.49 | 1.29 | 0.352 |  |
|  | CCI | 1.637 | 0.67 | 1.37 | 0.122 |  | CCI | 0.614 | 0.36 | 1.05 | 0.075 |  |
|  | **OS (overall survival)** | | | | | | | | | | |  |
|  | **Parameter** | **HR** | **95% CI** | | **p value** |  | **Parameter** | **HR** | **95% CI** | | **p value** |  |
|  |  |  | **Lower** | **Upper** |  |  |  |  | **Lower** | **Upper** |  |  |
|  | Baseline GHS | 0.977 | 0.96 | 0.99 | ***0.046*** |  | Baseline GHS | 0.966 | 0.95 | 0.99 | ***0.001*** |  |
|  | cT-stage | 1.914 | 0.62 | 2.56 | 0.982 |  | cT-stage | 1.426 | 0.50 | 4.06 | 0.506 |  |
|  | Gleason-grade biopsy | 1.037 | 0.53 | 1.90 | 0.412 |  | Gleason-grade biopsy | 1.293 | 0.87 | 1.91 | 0.198 |  |
|  | iPSA | 1.763 | 0.58 | 1.43 | 0.680 |  | iPSA | 1.133 | 1.04 | 1.23 | ***0.004*** |  |
|  | Age | 1.847 | 0.90 | 1.44 | 0.099 |  | Age | 0.922 | 0.71 | 1.20 | 0.548 |  |
|  | ASA-Score | 1.135 | 0.61 | 1.66 | 0.980 |  | ASA-Score | 1.085 | 0.86 | 1.36 | 0.483 |  |
|  | CCI | 0.610 | 0.03 | 13.96 | 0.757 |  | CCI | 1.416 | 0.85 | 2.37 | 0.184 |  |
|  |  |  |  |  |  |  |  |  |  |  |  |  |

**Suppl. Table. 3 Subgroup analysis of favourable and unfavourable intermediate risk PCa** Multivariable cox regression analysis regarding the endpoint MFS (metastasis-free survival), BRFS (biochemical recurrence free survival) and OS (overall survival)
