# Supplementary material for: Optimizing risk stratification for intermediate-risk prostate cancer – the prognostic value of baseline health-related quality of life
Source: World J Urol. 2024 Oct 20;42(1):585. doi: 10.1007/s00345-024-05298-2 (PMC11491415; doi:10.1007/s00345-024-05298-2)
Supplement: Supplementary file 6 — Supplementary Material 6 [file 345_2024_5298_MOESM6_ESM.docx]

**A**

|  | **MFS (metastasis free survival)** | | |  |
| --- | --- | --- | --- | --- |
|  | **Parameter** | **Chi-squared** | **p value** |  |
|  | Baseline GHS | 1.225 | 0.27 |  |
|  | Favourable intermediate risk [y/n] | 3.476 | 0.16 |  |
|  | cT-stage | 0.954 | 0.33 |  |
|  | Gleason-grade biopsy | 0.031 | 0.86 |  |
|  | iPSA | 1.556 | 0.21 |  |
|  | Age | 0.044 | 0.83 |  |
|  | ASA-Score | 0.636 | 0.43 |  |
|  | CCI | 0.609 | 0.12 |  |
|  | **Global** | 4.675 | 0.59 |  |
|  |  |  |  |  |

**B**

|  | **BRFS (Biochemical recurrence free survival)** | | |  |
| --- | --- | --- | --- | --- |
|  | **Parameter** | **Chi-squared** | **p value** |  |
|  | Baseline GHS | 0.008 | 0.93 |  |
|  | Favourable intermediate risk [y/n] | 4.141 | 0.42 |  |
|  | cT-stage | 0.437 | 0.51 |  |
|  | Gleason-grade biopsy | 7.027 | 0.28 |  |
|  | iPSA | 5.716 | 0.12 |  |
|  | Age | 0.135 | 0.71 |  |
|  | ASA-Score | 1.448 | 0.23 |  |
|  | CCI | 0.954 | 0.41 |  |
|  | **Global** | 10.026 | 0.12 |  |
|  |  |  |  |  |

**C**

|  | **OS (overall survival)** | | |  |
| --- | --- | --- | --- | --- |
|  | **Parameter** | **Chi-squared** | **p value** |  |
|  | Baseline GHS | 0.303 | 0.58 |  |
|  | Favourable intermediate risk [y/n] | 0.001 | 0.98 |  |
|  | cT-stage | 1.850 | 0.17 |  |
|  | Gleason-grade biopsy | 0.501 | 0.48 |  |
|  | iPSA | 1.751 | 0.19 |  |
|  | Age | 1.985 | 0.16 |  |
|  | ASA-Score | 0.237 | 0.63 |  |
|  | CCI | 0.413 | 0.21 |  |
|  | **Global** | 5.752 | 0.45 |  |
|  |  |  |  |  |

**Suppl. Table 4.** Schoenfeld residuals testing assumptions for Cox regression models
