# Supplementary material for: Optimizing risk stratification for intermediate-risk prostate cancer – the prognostic value of baseline health-related quality of life
Source: World J Urol. 2024 Oct 20;42(1):585. doi: 10.1007/s00345-024-05298-2 (PMC11491415; doi:10.1007/s00345-024-05298-2)
Supplement: Supplementary file 7 — Supplementary Material 7 [file 345_2024_5298_MOESM7_ESM.docx]

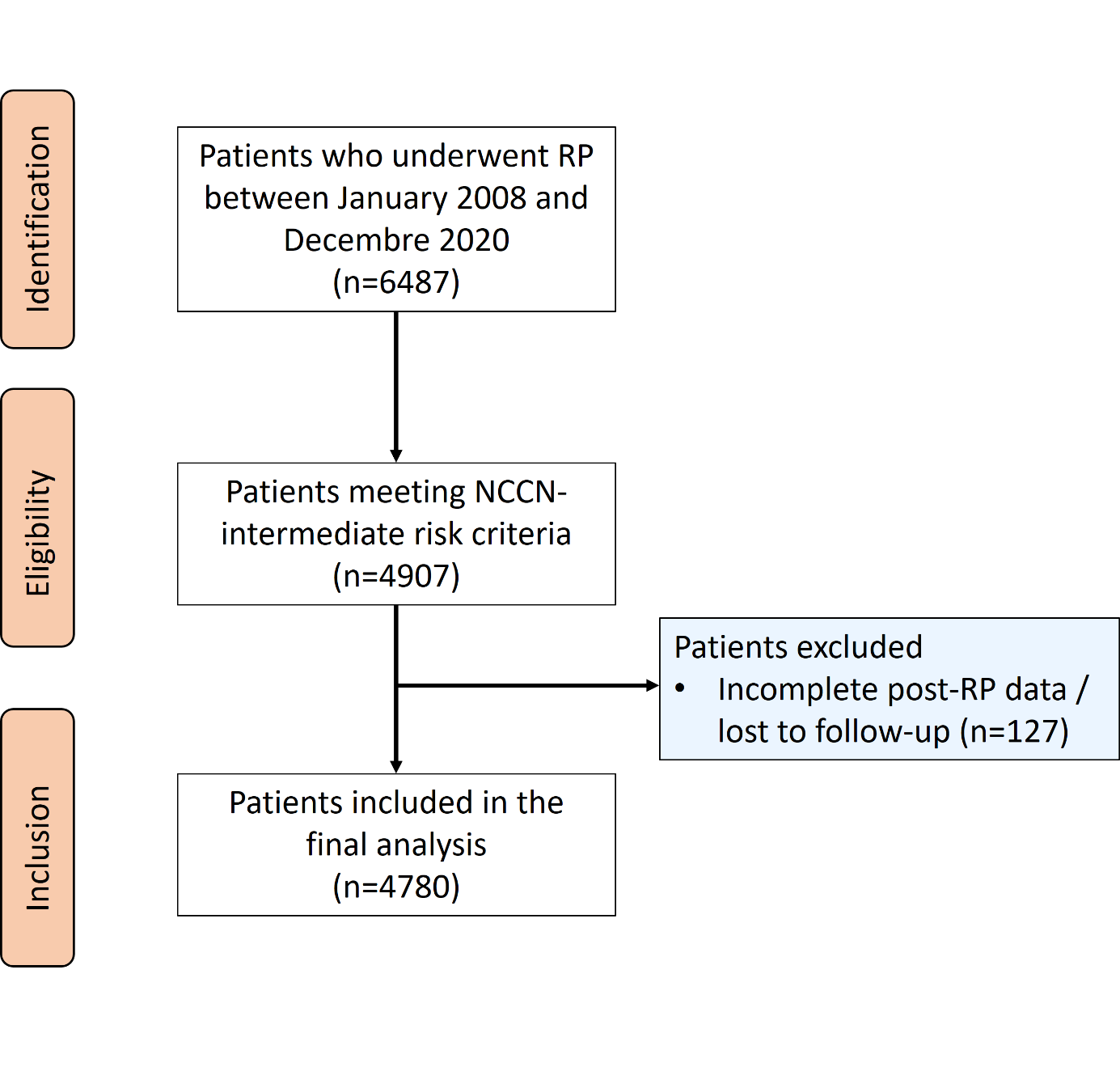
**Suppl. Figure 1** Flow chart summarizing the inclusion process for eligible subjects of the current study (RP = radical prostatectomy; PC = prostate cancer; NCCN = National Comprehensive Cancer Network).
