# Supplementary material for: Optimizing risk stratification for intermediate-risk prostate cancer – the prognostic value of baseline health-related quality of life
Source: World J Urol. 2024 Oct 20;42(1):585. doi: 10.1007/s00345-024-05298-2 (PMC11491415; doi:10.1007/s00345-024-05298-2)
Supplement: Supplementary file 8 — Supplementary Material 8 [file 345_2024_5298_MOESM8_ESM.docx]

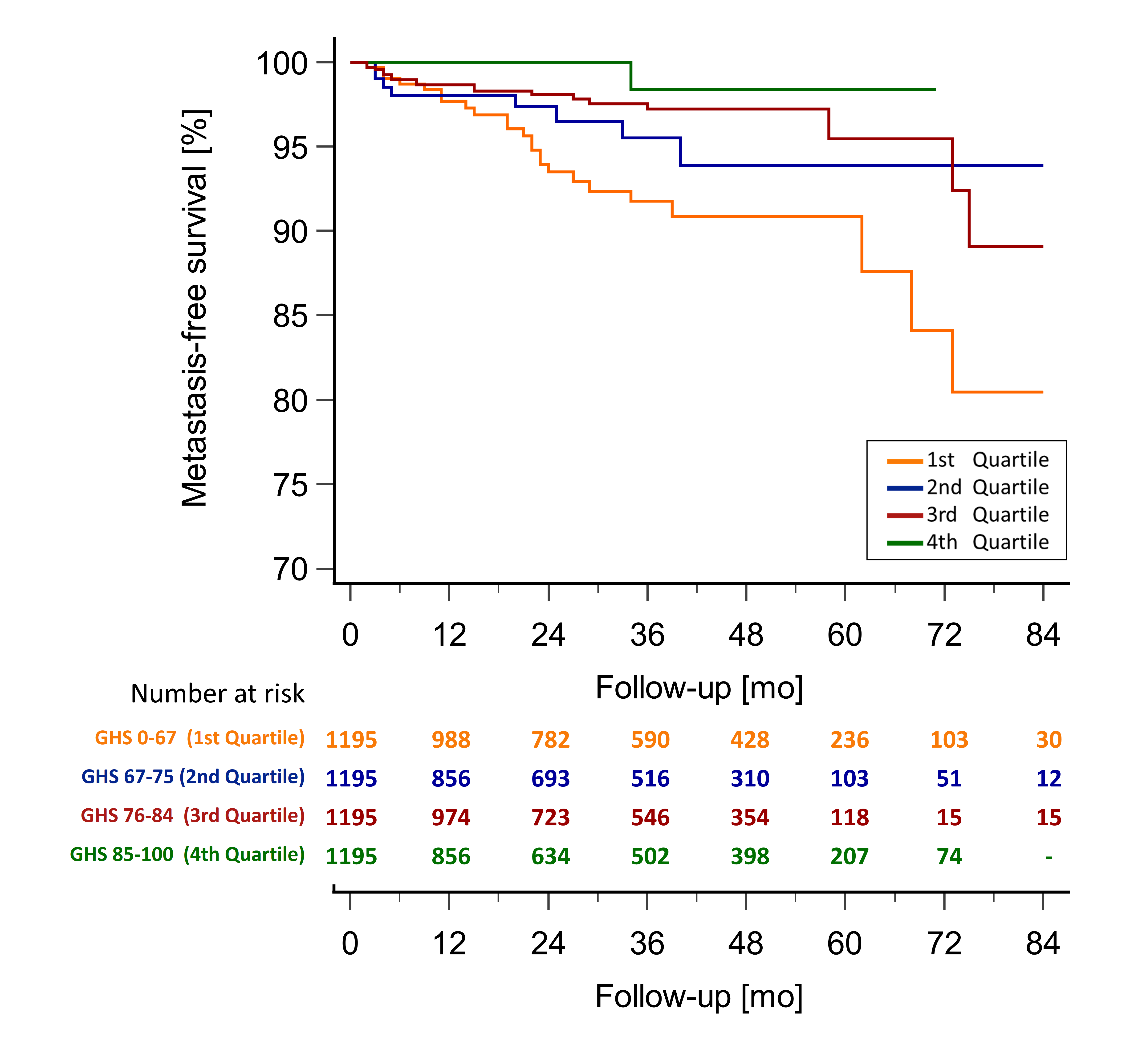


**Suppl. Figure 2.** Freedom from metastasis stratified by preoperative baseline EORTC QLQ-C30 global health status (GHS)
